# Observational constraints on the process and products of Martian serpentinization

**DOI:** 10.1126/sciadv.add8472

**Published:** 2023-02-03

**Authors:** Benjamin M. Tutolo, Nicholas J. Tosca

**Affiliations:** ^1^ University of Calgary, Department of Geoscience, Calgary, AB, Canada.; ^2^ University of Cambridge, Department of Earth Sciences, Cambridge, UK.

## Abstract

The alteration of olivine-rich rocks to serpentine minerals, (hydr)oxides, and aqueous hydrogen through serpentinization is long thought to have influenced the distribution of habitable environments on early Mars and the evolution of the early Martian hydrosphere and atmosphere. Nevertheless, the planetary importance of Martian serpentinization has remained a matter of debate. To constrain the process and products of Martian serpentinization, we studied serpentinized iron-rich olivines from the 1.1-billion-year Duluth Complex. These data indicate that serpentinized iron-rich olivine would have been accompanied by a fivefold increase in hydrogen production relative to serpentinized terrestrial mantle peridotites. In contrast to previous expectations, this style of serpentinization yields hisingerite as the dominant iron serpentine mineral at comparatively low temperature and pH, consistent with meteorite mineralogy and in situ rover data. The widespread occurrence of oxidized iron-bearing phyllosilicates in highly magnetized regions of the Martian crust supports the hypothesis that serpentinization was more pervasive on early Mars than currently estimated.

## Teaser

Observations suggest serpentinization on Mars provided environments, ingredients, and fuel for origin and subsistence of life.

## INTRODUCTION

Serpentinization, the water-driven alteration of olivine-rich rocks, has played an integral role in the long-term evolution of Earth’s surface environments (*1*). As a consequence, serpentinization is regarded as a potentially major contributor to warming the surface of early Mars (*2*–*4*), as well as in controlling the long-term fate of the Martian hydrosphere (*5*–*7*), synthesizing organic carbon (*8*), and sustaining habitable conditions early in the planet’s history (*8*–*10*). Because olivine is widespread on the Martian surface (*11*) and highly reactive in the presence of water (*12*), many researchers have hypothesized that serpentinization would have been common when liquid water interacted with the mafic crust (*1*, *7*, *12*).

**Fig. 1.**
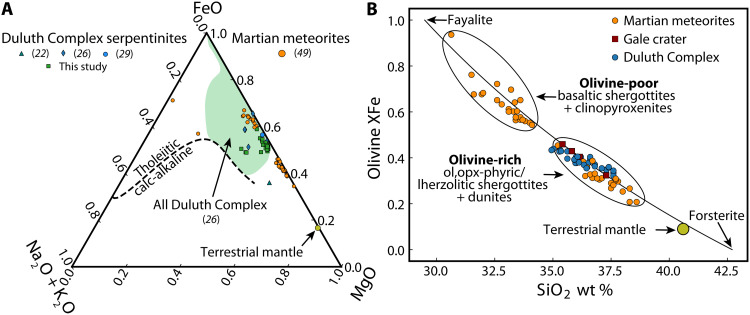
Comparison between (proto-)serpentinites of the Duluth Complex and available samples from Mars. (**A**) Alkali (Na_2_O + K_2_O)–iron (FeO)–magnesium (MgO) diagram comparing bulk geochemistry of Duluth Complex samples and Martian meteorites. The shaded field represents the range of Duluth Complex chemistry in samples compiled by (*26*), while the symbols represent either analyses of Martian meteorites or Duluth Complex samples with geochemistry that we classify as (proto-)serpentinites (see Materials and Methods) for use in subsequent analyses (Fig. 2). (**B**) Olivine XFe plotted against SiO_2_ weight % for olivines of the Duluth Complex, Mars (represented by Martian meteorites and samples analyzed by the Curiosity rover in Gale crater), and the terrestrial mantle (see Materials and Methods for references). The ideal solid solution between forsterite and fayalite is also plotted for reference. ol, olivine; opx, orthopyroxene.

Nevertheless, the influence of serpentinization on Martian habitability and early atmospheric evolution is largely unknown. Although the mineralogy of the ancient Martian crust records various interactions between olivine and water (*13*), the expected products of serpentinization are rare (*14*, *15*). In addition, serpentinization on Mars is commonly expected to have generated globally substantial quantities of H_2_, a potent greenhouse gas and energetic metabolic substrate (*2*). However, although serpentinization of mantle peridotite has been extensively studied on Earth, Martian basalts [~17 weight % (wt %) of FeO] have about double the Fe concentration of their terrestrial counterparts (7 to 10 wt % of FeO) (*5*), in turn, leading to olivine compositions that are distinctly Fe-rich compared to those derived from the terrestrial mantle (Fig. 1) (*11*). In addition, while it is widely acknowledged that the Fe content of the reactant olivine principally controls H_2_ generation (*12*), a lack of observational constraints from Fe-rich systems has left the rates and mechanisms of serpentinization of ferroan olivine poorly quantified. As a consequence, thermodynamic models extending serpentinization to more ferroan compositions have yielded conflicting results, with some predicting a decrease in H_2_ concentration with increasing olivine Fe content (*12*) and others predicting the opposite (*16*).

We addressed these uncertainties through a focused study of Fe oxidation and H_2_ generation during serpentinization of Fe-rich olivines in the 1.1 Ga Duluth Complex (MN, USA). Using a suite of geochemical techniques, we show that H_2_ is generated from ferroan olivine according to reaction mechanisms that ultimately lead to more H_2_ and, by extension, reduced carbon per mole of reactant olivine than terrestrial mantle peridotites. Our data also show that serpentinization of ferroan olivine yields distinct serpentine minerals compared to those derived from nearly all other serpentinizing systems on Earth. These minerals have not been targeted by previous orbital spectroscopic surveys yet represent a common feature of in situ mineralogical data and in meteorites (*17*–*19*). Thus, serpentinization of the Martian crust is likely to have been underestimated by orbital spectroscopic surveys to date, a conclusion supported by reanalysis of current orbital and geophysical datasets. By refining current geochemical and mineralogical criteria for the identification of ancient serpentinizing systems on Mars, these results directly inform ongoing and planned investigations of olivine-rich rocks by the Perseverance rover at Jezero crater, as it explores the largest concentration of olivine-rich rocks identified within the Martian crust.

## RESULTS

### Duluth Complex serpentinites provide a rare window into Martian serpentinization

Much of our current understanding of serpentinization is derived from studies of altered terrestrial mantle peridotites (*20*). However, in marked contrast to the terrestrial upper mantle, the Martian crust is largely tholeiitic in composition (*21*) and, in bulk, may be classified as an olivine metagabbro containing varying proportions of olivine, pyroxene, and plagioclase (*5*). Relative to known serpentinized protoliths on Earth, the 1.1-Ga Duluth Complex offers a close compositional analog to the Martian crust; it was emplaced over multiple intrusive events with layers and units forming a continuum between mafic and ultramafic lithologies (fig. S1). Despite local mineralogical and geochemical variation (*22*), Duluth Complex rocks are also tholeiitic on average, with generally low alkali (Na_2_O + K_2_O) contents and FeO and MgO concentrations that are consistent with those of the Martian meteorites (Fig. 1A). The ultramafic rocks, i.e., those that are prone to H_2_-generating serpentinization reactions and subsequent hydrocarbon production, rather than simple hydration reactions, contain as much as 80% or more modal olivine (*23*) with the balance typically made up of plagioclase, clinopyroxene, biotite, and oxides in the order of decreasing abundance (*22*). Collected chemistries of serpentinized ultramafic rocks from the Duluth Complex have a mean *X*_FeO_ = 0.55 ± 0.05 [where *X*_FeO_ = FeO/(FeO + MgO), in weight %], which is identical, within error, to that for the analyzed Martian meteorites [*X*_FeO_ = 0.52 ± 0.14 (1σ)] and much greater than *X*_FeO_ of the terrestrial mantle (0.18) (*24*). This similarity in chemistry suggests that serpentinites of the Duluth Complex provide a rare and accessible glimpse of Martian serpentinization reactions.

As expected from their bulk compositions, Duluth Complex and Martian olivines are also similar in composition and invariably more ferroan than terrestrial mantle olivine (Fig. 1B). Analysis of Martian meteorites has delineated two populations of olivines; olivine occurring in olivine-poor clinopyroxenites and basaltic shergottites, which have more ferroan compositions (*X*_Fe_ ≳ 0.5), and olivines of the generally more olivine-rich meteorites (the olivine- and olivine-orthopyroxene-phyric and lherzolitic shergottites and dunites), which have more magnesian compositions (*X*_Fe_ = 0.21 to 0.46). Olivines analyzed by the CheMin x-ray diffraction (XRD) instrument aboard the Curiosity rover (Fig. 1b) and spectral mapping of olivine occurrences with *X*_Fe_ = 0.32 to 0.61 (*11*) also fall into this latter population. Compositions of olivines of the Duluth Complex overlap with nearly the entire compositional range of olivines in olivine-rich Martian rocks (Fig. 1B). Even the most magnesian of the Martian olivines contain more than double the Fe content of terrestrial mantle olivine (*X*_Fe_ = 0.09). This comparison suggests that studies based on serpentinization of terrestrial mantle cannot provide mechanistic insight into H_2_ production during Martian serpentinization and likely underestimate the fluxes of H_2_ from these reactions on Mars.

Fluid inclusion analyses suggest that hydrothermal alteration of the Duluth Complex occurred at temperatures from 700° to <200°C, driven by NaCl/CaCl_2_-bearing fluids derived from melt volatilization, metasedimentary country rocks (*25*), and/or meteoric fluids. The higher-temperature phase of hydrous alteration is indicated by amphibole in some rocks (*26*), but generally, the most highly altered rocks from the Duluth Complex are those with the highest protolith olivine abundances (*23*). In these rocks, serpentine generally occurs as fractures in olivine (fig. S2) (*25*, *27*–*29*), although massive replacement of picrite or peridotite layers is also commonly observed (*26*). The lower temperature limit of olivine stability decreases with increasing fayalite content, such that, while the upper temperature limit for serpentinization of terrestrial mantle peridotite is around 350°C, the upper temperature limit for serpentinization of pure fayalite is closer to 200°C (fig. S3A) (*12*, *30*). For this reason and the observed hydrothermal precipitation of Fe-enriched olivine, Evans *et al*. (*27*) speculated that Duluth Complex serpentinization occurred at ~200°C, which was validated with subsequent thermodynamic calculations (*28*). This low temperature would suggest that serpentinization should have been accompanied by minimal magnetite production (*31*), but, as we show below, that is apparently not the case. Together, these results suggest that the lowest temperature, latest stage of hydrothermal alteration in the Duluth Complex, drove serpentinization and that understanding Martian serpentinization thus requires a renewed focus on lower-temperature serpentinization.

### Fe oxidation and H_2_ production during serpentinization of ferroan olivine

**Fig. 2.**
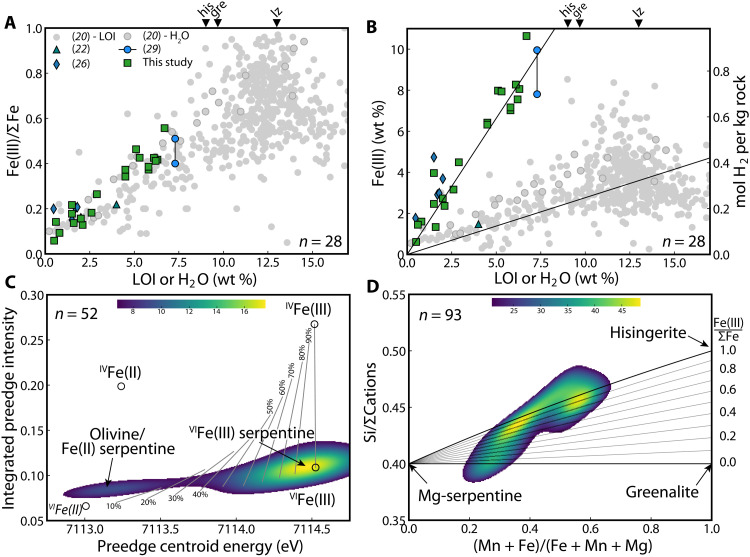
Iron oxidation and hydrogen generation during serpentinization of the Mars analog Duluth Complex. (**A**) Compilation of Fe oxidation states in serpentinites as a function of LOI or, when available, H_2_O weight % for Duluth Complex compared to a global compilation of terrestrial mantle serpentinites (*20*). (**B**) Oxidized Fe [Fe(III)] weight % as a function of LOI or, when available, H_2_O weight %. H_2_ generation per kilogram of rock was calculated by assuming the protoserpentinite contained exclusively reduced Fe(II). The H_2_O weight % values corresponding to hisingerite (his), greenalite (gre), and lizardite (lz) are denoted along the top axis in (A) and (B). (**C**) Kernel density plot of 52 x-ray absorption near-edge spectroscopy (XANES) measurements of Fe oxidation states in Duluth Complex samples. Labeled lines indicate the percentage of Fe(III) (**D**) Kernel density plot of 93 electron microprobe (EMP) analyses of Duluth Complex serpentines compared to end member serpentine values.

Although the Duluth Complex has been extensively sampled for mineral exploration purposes, few data relating H_2_ production to the extent of serpentinization exist (Fig. 2, A and B, and Materials and Methods). Thus, to quantify H_2_ production during serpentinization of Duluth Complex olivines, we performed bulk-rock geochemistry, Fe redox titrations, and H_2_O content determinations on 20 serpentinites specifically sampled to represent the varying degrees of serpentinization observed across the Complex, which range from minimal to nearly complete (*25*, *29*, *32*). Our new and collected bulk compositional analysis of serpentinized Duluth Complex rocks show that, as the nominally anhydrous protolith peridotite is progressively serpentinized, its water content [indicated by increasing H_2_O or loss on ignition (LOI) weight (wt) %] and proportion of oxidized Fe increase in accordance with studies of terrestrial mantle peridotites (Fig. 2A) (*20*, *33*). Although this relationship might suggest a close similarity between Duluth Complex serpentinites and those derived from the terrestrial mantle, the two systems differ markedly in the total amount of Fe oxidized as a function of serpentinization progress. Consistent with their much higher initial Fe content, serpentinized rocks from the Duluth Complex contain around five times higher Fe(III) by weight for a given degree of serpentinization; this directly translates to a factor of five increase in the H_2_ production over this interval (Fig. 2B). Because of their much higher Fe content, the canonical value of 13 wt % (i.e., the H_2_O content of the mineral lizardite) for “complete” serpentinization of terrestrial mantle rocks does not apply. Rather, because the Fe-serpentines hisingerite and greenalite contain much less water by weight—9.02 and 9.69 wt % of H_2_O, respectively—complete serpentinization of the much more ferroan Mars and Duluth olivines should correspond to these lower values. Regardless, a fivefold increase in H_2_ for any given degree of serpentinization of Duluth Complex and Martian rocks means that even a weakly serpentinized (20%) rock on Mars will produce as much H_2_ as a fully serpentinized terrestrial mantle peridotite.

Our data also show that H_2_ production in Duluth Complex and Martian serpentinites proceeds according to a fundamentally different mechanism compared to serpentinization of the terrestrial mantle. The dominant mechanism for Fe^3+^ accommodation in the serpentine structure during serpentinization of the terrestrial mantle is through ferri-Tschermaks [2 Fe^3+^ ↔ (MgSi)] substitution (*34*). This yields serpentines that are solid solutions between Mg-serpentine (e.g., lizardite and Mg_3_Si_2_O_5_OH_4_), greenalite (Fe^2+^_3_Si_2_O_5_OH_4_), and Mg-cronstedtite (Mg_2_Fe^3+^_2_SiO_5_OH_4_), with ~50% Fe(III) in octahedral [^VI^Fe(III)] coordination and ~50% Fe(III) in tetrahedral [^IV^Fe(III)] coordination, depending on the extent of other R^3+^ cations such as Al^3+^ in the serpentine structure (*34*). In contrast, our new Fe K-edge x-ray absorption near-edge spectroscopy (XANES) and compiled electron microprobe (EMP) analyses show that serpentines of the Duluth Complex incorporate Fe^3+^ not through ferri-Tschermaks substitution but rather through a vacancy-coupled Fe^3+^ substitution for Mg^2+^ in the serpentine octahedral site, leading to serpentines that are solid solutions among Mg-serpentine, greenalite, and hisingerite (Fe^3+^_2_Si_2_O_5_OH_4_) (Fig. 2,C and D). Unlike typical redox-specific XANES microanalyses of seafloor serpentinites, which plot in an upward-sloping trend toward a 50/50 ^IV^Fe(III)/^VI^Fe(III) mixture (*35*), our XANES analyses (Fig. 2C) plot in a flat trajectory toward ^VI^Fe(III), with negligible ^IV^Fe(III). Moreover, cation-sensitive EMP analyses (Fig. 2D) confirm that these serpentinization reactions lead to abundant Fe^3+^ incorporation into the serpentine structure (i.e., a trajectory toward the hisingerite end member) with only a minor proportion Fe(II)-bearing greenalite and little or no ^IV^Fe(III) incorporation. At the bulk scale, Mössbauer analyses indicate that around half of the oxidized Fe in the most serpentinized sample (i.e., highest LOI or H_2_O weight %) in our compilation is hosted within magnetite (fig. S4 and table S1); this holds true for the more oxidized [Fe(III)/ΣFe = 0.51] fracture zones [46% of Fe(III) in magnetite] and the less oxidized [Fe(III)/ΣFe = 0.40] matrix [49% of Fe(III) in magnetite]. The trend toward Mg-serpentine-greenalite-hisingerite solid solutions rather than ferri-Tschermaks substitution characteristic of terrestrial serpentinites is evidently related to both the protolith olivine composition and environmental parameters such as silica activity and pH (*27*, *28*, *33*).

The hisingerite and magnetite-forming Fe oxidation reactions are, at least in samples from the southern portion of the Duluth Complex, accompanied by a general trend toward Fe enrichment in serpentines relative to their protolith olivines, especially when considering the significant fraction of the Fe that is going toward magnetite production (*27*, *28*). This behavior reflects substantial Mg mobility during Martian serpentinization, which, in turn, implies highly acidic fluids (Fig. S3B) (*27*, *28*), and is thus quite unlike serpentinization of the terrestrial mantle where serpentines tend to be Mg-enriched, fluid pH tends to be alkaline, and Mg tends to be highly immobile (*36*). Although initial work (*27*) suggested that fluid acidity would have been related to sulfide mineralization in the surrounding Duluth Complex, subsequent efforts (*28*) concluded that it is more likely related to internal olivine-plagioclase buffering of pH, in a manner similar to the ultramafic-associated Rainbow hydrothermal vent field on the mid-Atlantic Ridge (*37*), in turn, suggesting that such acidic fluids may also be the drivers of serpentinization on Mars. Although our results demonstrate abundant H_2_ generation during Martian serpentinization, unraveling other aspects of fluid chemistry evolution will require independent constraints on governing variables such as water-rock ratio. Initial fluid composition has been shown to play a role in alteration mineralogy only at high (≫10) water-to-rock ratios (*30*, *34*), so our results are expected to provide a robust indication of serpentinization processes on early Mars, regardless of initial fluid composition.

## DISCUSSION

### Implications and tests of serpentinization-driven Martian habitability

Recent geophysical constraints on the structure of the Martian upper mantle (*38*) and elevated porosity of the Martian crust (*39*) suggest that serpentinizing fluids would have had continual access to fresh, Fe-rich olivine. Convection cells driven by enhanced crustal heat flow (*39*) would have both pulled surface water into the deep olivine-rich subsurface and delivered the H_2_-laden fluids back to the surface, just as they do throughout Earth’s ocean basins today (*36*, *37*). Using our results for H_2_ production during Martian serpentinization, we calculate that overcoming maximum estimated H_2_ escape rates on early Mars (*3*) would require a global average serpentinization advance rate of just 1 × 10^−7^ km/year (see Materials and Methods), well within the plausible range of ultramafic rock reaction front advance rates on Earth (10^−8^ to 10^−4^ km/year) (*40*–*42*). This suggests that active serpentinization under 5% of the Martian surface [cf. (*3*)] alone could feasibly outstrip H_2_ escape to space and lead to significant H_2_ accumulations in the early Martian atmosphere; coupling these H_2_ emissions with the likely, significant coupled fluxes of CH_4_ related to serpentinization processes and a CO_2_-rich atmosphere could easily have led to warm and wet conditions on ancient Mars (*2*–*4*). At the same time, if serpentinization reactions were confined to just a fraction of the Martian surface but still producing fluxes sufficient to overcome escape velocities, then significantly higher local concentrations of the ingredients and substrates for the origins and potential subsistence, respectively, of early life forms would be expected in these localized regions.

Our results also indicate that orbital investigations of the Martian surface have not targeted minerals likely to be derived from serpentinization of ferroan olivine. Orbital remote sensing data have been used to search for Mg-rich serpentine minerals such as lizardite and antigorite, under the assumption that serpentinized crust on Mars should be analogous in mineralogy to serpentinized terrestrial mantle peridotite (*14*, *15*). These minerals, distinguishable from infrared absorptions at 1.4 and 1.9 μm (from structurally bound OH and H_2_O), and a unique Mg-OH absorption between 2.10 and 2.12 μm (*15*) have rarely been found at high concentration within the exposed Martian bedrock, leaving the role of serpentinization on early Mars largely unresolved (*14*, *15*). In contrast, hisingerite, the dominant serpentine produced from serpentinization of ferroan olivine, exhibits the same absorptions at 1.4 and 1.9 μm and a distinct Fe(III)-OH absorption at 2.28 μm; these spectral characteristics are identical to Fe(III)-rich smectites such as nontronite (*43*, *44*), the dominant phyllosilicate commonly interpreted to be present in high concentration in ancient Martian bedrock (*45*, *46*).

**Fig. 3.**
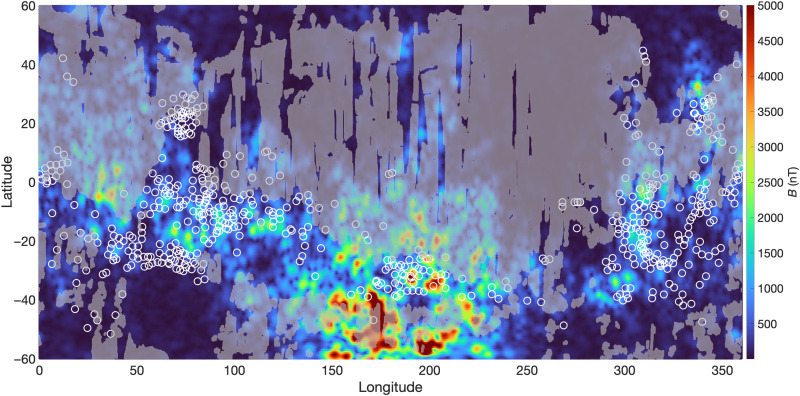
Magnetic field map (0.5° resolution) (*45*). Map color intensities have been rescaled emphasize the midvalues. Max values of the map, which occur in the highly magnetized region to the far south, reach ~11,000 nT. Circles overlain are detections of Fe(III) layer silicates that are spectrally indistinguishable from hisingerite (*42*). These identifications were grouped according to the position of the ~2.28-μm absorption, which is relatively sensitive to Fe/Mg content. Although they were interpreted as smectites, all plotted detections would be spectrally indistinguishable with hisingerite. Gray shaded regions correspond to highly dust-covered regions (OMEGA dust index > 1) (*46*), which has been shown to decrease the frequency of spectroscopic identifications of hydrous minerals (*43*).

If nontronite and hisingerite cannot be distinguished in orbital remote sensing datasets, then previous global surveys reporting the presence of nontronite (based on the 2.28-μm band with minor fluctuations in wavelength according to minor Mg substitution) provide a maximum estimate of the global distribution of serpentinized bedrock exposures on Mars. Many regions characterized by high concentrations of Fe(III)-phyllosilicates (interpreted as nontronite) are pervasive across the ancient southern highlands (*45*), and many coincide with anomalously high crustal magnetic field strength, indicating that magnetite may also be an important crustal constituent (Fig. 3). Consistent with these predictions, hisingerite of preterrestrial origin has been identified in some nakhlite meteorites (*19*), and powder XRD and fluorescence data indicate that the mineral may be a pervasive constituent of Gale Crater sedimentary rocks (*17*, *18*, *47*). Together, our data, in combination with orbital spectroscopic and geophysical data, support the hypothesis that large portions of the ancient Martian crust have been serpentinized and potentially yielded globally significant quantities of H_2_.

H_2_ fluxes from serpentinizing systems on early Mars would have provided environments, ingredients, and fuel for the origin and potential early evolution of microbial life. H_2_-driven greenhouse enhancements would have stabilized liquid water on the planet’s surface; H_2_-driven prebiotic chemistry would have enabled formation of vital components of primordial cells; and H_2_-driven carbon fixation could have acted as an energy source for any nascent organisms. Although serpentinization’s potential contribution to Martian planetary habitability has been recognized for some time, our quantitative examination of Duluth Complex serpentinites provides new mineralogical criteria for serpentinization of the Martian crust and suggests that much of the ancient Martian surface, including Jezero Crater, may preserve remnants of these climatically and astrobiologically significant systems.

## MATERIALS AND METHODS

### Comparison of Martian meteorite and Duluth Complex bulk geochemistry

To explore the potential for Duluth Complex serpentinites [fig. S1; mapped in (*48*)] to yield insight into Martian serpentinization, we compiled bulk geochemical analyses of Martian meteorites (table S2) (*49*) and compared them against analyses of samples from the Duluth Complex (table S3). The range of geochemical compositions of the Duluth Complex was established by Severson (*26*), who compiled 520 complete analyses of exploration drill core samples. These data were plotted in an alkali (Na_2_O + K_2_O)–iron (FeO)–magnesium (MgO) diagram and outlined to establish the range of compositions represented by the shaded field in Fig. 1A. The primitive terrestrial mantle composition presented by McDonough and Rudnick (*50*) was also plotted for comparison.

To examine H_2_ generation during serpentinization reactions represented in this compiled dataset, we screened the analyses to just include those that were olivine-rich (30 to 45 wt % of SiO_2_), included explicit determination of Fe redox state (i.e., both FeO and Fe_2_O_3_) and LOI or, preferably, water content (H_2_O^+^), and contained minimal TiO_2_ (<1.5 wt %). Notably, this last restriction was required, because much of the geochemical data from the Duluth Complex was focused on finding economically viable oxide deposits and cumulate enrichments of TiO_2_ in the form of titanomagnetite [Fe^2+^(Fe^3+^,Ti)_2_O_4_] (*26*) imply a protolith with nonneglible primary Fe(III), thereby complicating the quantification of Fe(III), and hence H_2_, generation during serpentinization. Although this 1.5% TiO_2_ threshold is arbitrary, it has the intended effect of screening out analyses with low H_2_O content and high Fe(III)/Fe(tot). Given these requirements, the Severson (*26*) dataset was reduced to just five applicable analyses; this resultant dataset was supplemented by one applicable analysis presented by Weiblen and Morey (*22*) and analyses of our own samples obtained from the Bardon Peak peridotite, chosen to illustrate the full range of degrees of serpentinization in the Duluth Complex. One sample, initially analyzed for bulk geochemistry by Tutolo *et al*. (*29*) was reanalyzed for the present study via Mössbauer spectroscopy. To assist in delineating redox heterogeneity effects and the portioning of Fe(III) between magnetite and other phases in the sample, this sample was analyzed once each for the “matrix” and “fracture” portions described by Tutolo *et al*. (*29*) by drilling these sections of the sample with a dental drill. An additional 20 analyses of samples obtained from the Bardon Peak outcrop in May 2019 were analyzed for the present study by Actlabs (Ancaster, Ontario, Canada) for bulk geochemistry (including total Fe as Fe_2_O_3_) by x-ray fluorescence (XRF), moisture content (H_2_O^−^) via gravimetry, water content (H_2_O^+^) via infrared spectroscopy, and FeO content via a modified Wilson titration method. The resultant values of FeO and Fe_2_O_3_(tot) were then used to determine Fe(III)/Fe(tot).

The collected Duluth Complex protolith assemblages would be classified as peridotite, troctolite, and/or olivine gabbro, all consistent with olivine-rich geology expected for protoserpentinites on Mars. Together, the 28 analyzed serpentinites cover a range of H_2_O/LOI contents from near zero (0.5 wt %) to 7.29 wt % and Fe(III)/Fe(tot) from ~5 to ~56%, suggesting that they represent the full range of extents of serpentinization present in the Duluth Complex. From these collective analyses and the assumption that all Fe in the protolith was present as Fe(II), we calculated the amount of H_2_ produced per kilogram of rock according to [cf. Mayhew and Ellison (*20*)]

$$\mathrm{FeO}+0.5\;H_{2}O=0.5\;H_{2}+0.5\;{\mathrm{Fe}}_{2}O_{3}$$ (1)

The resultant values for Fe(III)/Fe(tot) and H_2_ produced per kilogram of rock versus H_2_O weight % (when available) or LOI (when H_2_O weight % was unavailable) for Duluth Complex serpentinites were plotted against equivalent values for terrestrial mantle serpentinites compiled by Mayhew and Ellison (*20*) for comparison.

### Comparison of Martian, Duluth Complex, and terrestrial mantle olivines

EMP analyses of olivines in Martian meteorites were compiled from various sources and supplemented with Gale Crater olivine compositions empirically calculated from CheMin XRD analyses performed by the Mars Science Laboratory Curiosity rover. For comparison to the range of olivine compositions observed in Martian rocks, we also compiled EMP analyses of igneous olivines in peridotites of the Duluth Complex. To capture the variation in composition throughout the complex, we compiled data for the Longnose peridotite and the Bardon Peak peridotite and nearby rocks in the southern portion of the complex and the Kawishiwi intrusion in the northern portion of the complex. For comparison, we also added data for San Carlos olivine to the compilation (*51*). The compiled olivine analyses were screened to remove any analyses that summed to <98 or >102% and are presented with accompanying references in table S4. The pyrolite software package (*52*) was used to calculate *X*_Fe_ from the measured concentrations of SiO_2_, MgO, FeO, and, where available, NiO and MnO or, in the case of the Gale Crater samples and ideal olivine compositional line, used to calculate oxide concentrations from values of *X*_Fe_.

### Thermodynamic exploration of Fe-rich olivine serpentinization reactions

To delineate the geochemical conditions under which serpentinization produces hisingerite and magnetite at the expense of Fe-rich olivine, we conducted a series of thermodynamic calculations using the Geochemist’s Workbench Release 17.0.0 using a custom thermodynamic database created with the PyGeochemCalc software package (*53*). We first calculated the relative stability of Fe-enriched olivines relative to olivine of the terrestrial mantle (which is ~90% forsterite/90% fayalite; i.e., it is Fo90) to demonstrate the lower temperatures of serpentinization on Mars relative to Earth (fig. S3A). Subsequently, we calculated the relative stability of the three end members of Martian serpentinization [Mg-serpentine (here represented by lizardite), greenalite, and hisingerite] to demonstrate the elevated activities of H^+^ (i.e., the reduced pH) under which hisingerite is stable (fig. S3B).

### X-ray absorption near-edge spectroscopy

XANES data were acquired in fluorescence mode on polished 150-μm-thick section samples angled at 45° to the incident beam at Diamond Light Source beamline i18. Before analysis, beam energy was calibrated using Fe foil, and a 0.1-mm Al filter was placed in the beam path to prevent sample damage, which was verified by comparison of repeated spot analyses. Serpentines in the Duluth Complex sample were chosen for analysis by identifying them in high-resolution (5-μm) and low-resolution (10-μm) XRF mapping and then analyzed via a 10-μm beam. Analyses were acquired over the energy range 7000 to 7300 eV: 7000 to 7100 eV at 5-eV steps, 7100 to 7105 eV at 1-eV steps, 7105 to 7120 eV at 0.1-eV steps, 7120 to 7140 at 1-eV steps, 7140 to 7200 eV at 2-eV steps, and 7200 to 7300 eV at 5-eV steps, with count times of 1 s between 7000 and 7105 eV, 5 s between 7105 and 7120 eV, 2 s between 7120 and 7140 eV, and 1 s between 7140 and 7300 eV. The resultant spectra were processed using techniques outlined by Zhang and Tutolo (*54*). Specifically, the spectra were processed by first subtracting background absorbance acquired between 7000 and 7050 eV and then normalizing the intensity to the average absorbance measured above 7250 eV. Pre-edge features were extracted by subtracting a three-term Gaussian function fit to the normalized absorbance measured above and below the features, whose positions changed depending on the bulk oxidation state of the analyzed sample. The preedge features were then fit using four fixed-width (1.5 eV) Gauss-Lorentz components and included in table S5. The resultant values were compared to fits of spectra acquired on ^[VI]^Fe(II)-bearing San Carlos olivine [National Museum of Natural History (NMNH) 111312-44], ^[IV]^Fe(II)-bearing staurolite (NMNH 117183), ^[VI]^Fe(III)-bearing andradite (NMNH 166396), and ^[IV]^Fe(III)-bearing plagioclase (NMNH 115900) provided by the Smithsonian Institution NMNH. These materials represent the end members in a variogram interpretation of oxidation state (*35*, *54*); identical processing of the average spectra at 10% intervals between the standards (e.g., the sum of 10% San Carlos olivine spectrum and 90% of the andradite spectrum) was also performed to provide an indication of the variation in oxidation state between end members, which can be nonlinear. Recent evaluations suggest an estimated uncertainty of 15 to 20% absolute on the resulting value Fe(III)/ΣFe (*54*).

Because serpentine often occurs as micrometer-scale veinlets in and around olivine grains, the 10-μm beam size and slightly larger illuminated area resulting from the angle of the sample with respect to the incident beam likely led some XANES analyses to be mixtures of olivine, serpentine, magnetite, and/or neighboring plagioclase. This spectral mixing, combined with potential spectral shifts related to orientation effects, suggests that evaluating the entire dataset enables more robust conclusions than focusing on individual spectra [cf. Ellison *et al*. (*35*)]. To do this, we used pyrolite (*52*) to compute kernel density estimates (KDEs) of the aggregated XANES data and plotted the resultant KDEs as a heatmap overlayed on the variogram computed from the XANES model compounds discussed above [cf. Ellison *et al*. (*35*)]. To facilitate visualization of the relevant end members in the aggregate data, we fixed the lower limit for the KDE plotted in Fig. 2 to 40% of the maximum.

### EMP analyses

EMP analyses of Duluth Complex serpentines were collected from the literature, and the resultant dataset was screened to remove points with anomalously high Si, Ca, and/or Al indicative of mixing with other phyllosilicates such as smectite and/or chlorite. The remaining 93 analyses were then converted to a seven-oxygen formula using pyrolite (table S6) (*52*). From the resultant mineral formulas, we calculated (Mn + Fe)/(Fe + Mn + Mg) and Si/ΣCations, where ΣCations is the sum of the cation atoms per formula unit for all cations for which analyses were available (Si, Al, Fe, Mn, Mg, Ni, and/or Ca) but is dominated by Si, Fe, Mn, and Mg, for comparison against values corresponding to the end member serpentines: Mg-serpentine [i.e., antigorite, lizardite, and/or chrysotile Mg_3_Si_2_O_5_(OH)_4_], hisingerite [Fe^3+^_2_Si_2_O_5_(OH)_4_], and greenalite [Fe^2+^_3_Si_2_O_5_(OH)_4_] and computed the KDE of the resultant dataset. To facilitate visualization of end members in the aggregate data, we fixed the lower limit for the KDE plotted in Fig. 2 to 35% of the maximum.

### Flux calculations

We calculate the degree of serpentinization required to overcome the maximum estimated hydrogen escape rate of 6.6 × 10^11^ molecules/cm^2^ per second (*3*) by dividing this value by Avogadro’s number (6.02 × 10^23^ molecules/mol), the maximum H_2_ production rate observed in our Duluth Complex dataset (0.95 mol of H_2_/kg of rock), and the density of “fresh” Martian peridotite (~3500 kg/m^3^) (*38*) and converting to units of kilometer per year. This yields a value of 1 × 10^−7^ km/year, which compares favorably with the range of serpentinization advance rates (10^−8^ to 10^−6^ km/year) validated against experimental, modeling, and field parameterizations (*40*), the 10^−4^ km/year reaction front advance rate derived from measurements of the Caledonian Köli Nappe (Northern Norway) (*41*), and the plausible serpentinization rates derived from permeability measurements (10^−7^ to 10^−6^) (*42*).
