# Supplementary material for: Observational constraints on the process and products of Martian serpentinization
Source: Sci Adv. 2023 Feb 3;9(5):eadd8472. doi: 10.1126/sciadv.add8472 (PMC9897658; doi:10.1126/sciadv.add8472)
Supplement: Supplementary file 1 — Figs. S1 to S4 Table S1 [file sciadv.add8472_sm.pdf]

Science Advances
AAAS

Supplementary Materials for

Observational constraints on the process and products of Martian serpentinization

Benjamin M. Tutolo and Nicholas J. Tosca

Corresponding author: Benjamin M. Tutolo, benjamin.tutolo@ucalgary.ca

Sci. Adv. **9**, eadd8472 (2023)
DOI: 10.1126/sciadv.add8472

The PDF file includes:

Figs. S1 to S4
Table S1
Legends for tables S2 to S6

Other Supplementary Material for this manuscript includes the following:

Tables S2 to S6

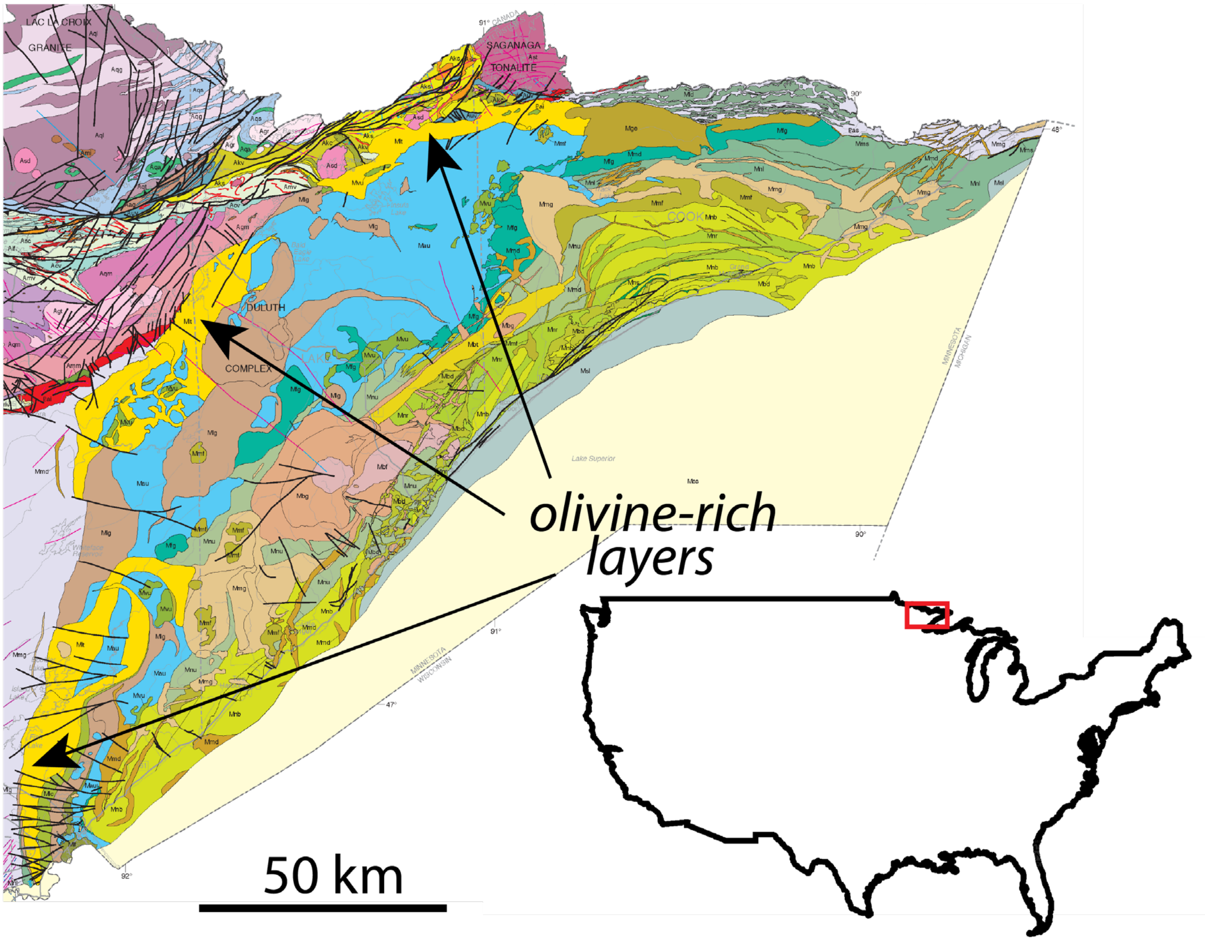

Figure S1 Geologic map of the Duluth Complex showing the locations of olivine-rich layers (modified from (*48*)).

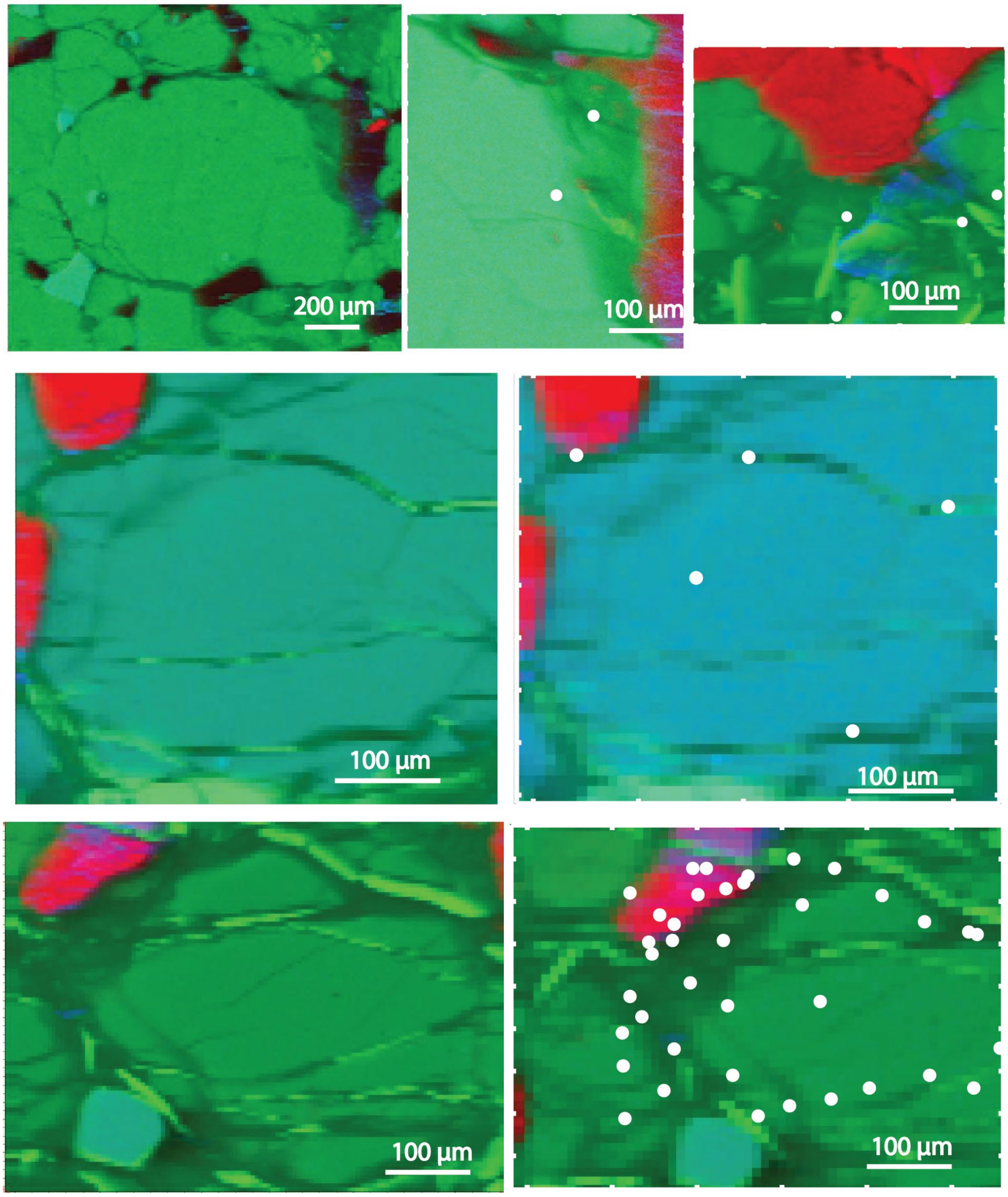

Figure S2 X-ray Fluorescence (XRF) maps of Duluth Complex samples showing locations of XANES analyses. Maps without points are higher-resolution versions of the adjacent maps with points. Colors are representative of the sample chemistry where Red = Ca, Green = Fe, and Blue = Mn.

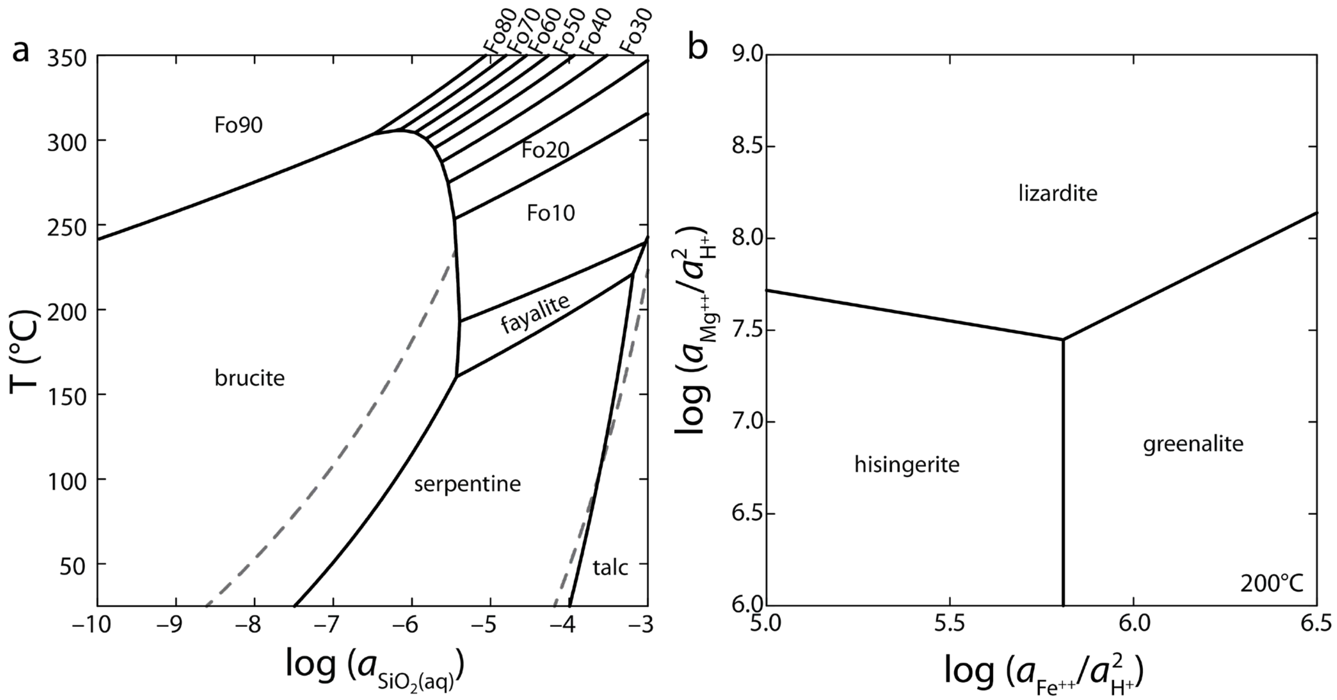

Figure S3 Thermodynamic exploration of Fe-rich olivine serpentinization reactions. **a** Temperature-activity diagram illustrating the increased stability of Fe-rich olivines relative to serpentinization products. brucite (Fe-brucite – brucite), serpentine (lizardite – greenalite), and talc (talc-minnesotaite) fields denote the stability of solid solutions. Solid lines indicate the equilibrium silica activity between Mg end-members and dashed lines indicate equilibrium silica activity between Fe(II) end-members. Redox transformation are not considered. **b** Activity diagram for the three end-members of Martian serpentinization in the presence of magnetite, showing hisingerite formation is favored in low pH (i.e., elevated activity of H^+), Fe-bearing serpentinizing fluids. Assuming Fe^{++} activity = 10^{-3}, the hisingerite field begins at pH ≈ 4.4. Abbreviation Fo = forsterite; number indicates relative percentage of forsterite with the remainder comprised of fayalite.

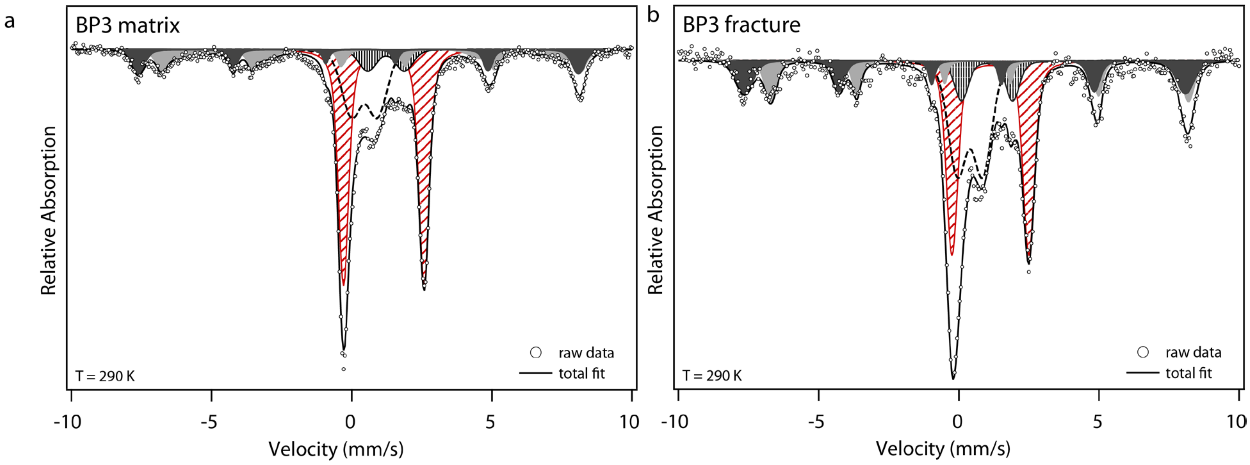

Figure S4 Mossbauer analyses of **a** "matrix" and **b** "fracture" zones analyzed by (*29*). Raw data is represented by circles and the components of the fit are represented by shaded curves. Goodness of fit, as indicated by calculated χ^2 , is 1.15 and 0.82 for **a** and **b**, respectively

Table S1 Mössbauer analyses of Fe(III) partitioning in Bardon Peak Sample from (*29*)

	**Fe(III)/ΣFe (%)**	**Fe(III) in mgt / ΣFe**
Matrix	0.40	0.49
Fracture	0.51	0.46

Captions for supplemental files

Table S2 Meteorite geochemistry compiled by (*49*) plotted in this study. References are after (*49*).

Table S3 Geochemical analyses of Duluth Complex (proto-)serpentinites

Table S4 Geochemical analyses of Martian and terrestrial olivines

Table S5 XANES analyses of serpentines and surrounding minerals from the Duluth Complex

Table S6 Electron microprobe analyses of Duluth Complex serpentines
